# Odorant receptors of *Drosophila* are sensitive to the molecular volume of odorants

**DOI:** 10.1038/srep25103

**Published:** 2016-04-26

**Authors:** Majid Saberi, Hamed Seyed-allaei

**Affiliations:** 1 School of Cognitive Sciences, Institute for Research in Fundamental Sciences (IPM), Tehran, Iran

## Abstract

Which properties of a molecule define its odor? This is a basic yet unanswered question regarding the olfactory system. The olfactory system of *Drosophila* has a repertoire of approximately 60 odorant receptors. Molecules bind to odorant receptors with different affinities and activate them with different efficacies, thus providing a combinatorial code that identifies odorants. We hypothesized that the binding affinity of an odorant-receptor pair is affected by their relative sizes. The maximum affinity can be attained when the molecular volume of an odorant matches the volume of the binding pocket. The affinity drops to zero when the sizes are too different, thus obscuring the effects of other molecular properties. We developed a mathematical formulation of this hypothesis and verified it using *Drosophila* data. We also predicted the volume and structural flexibility of the binding site of each odorant receptor; these features significantly differ between odorant receptors. The differences in the volumes and structural flexibilities of different odorant receptor binding sites may explain the difference in the scents of similar molecules with different sizes.

We know which properties of visible light are measured by our eyes, and we also know how our eyes process light. This knowledge has assisted in the production of cameras and displays. Unfortunately, we do not have the same knowledge regarding olfaction. We do not know the relationship between the molecular properties of a stimulus and the sensory response (i.e., the quality of a smell).

Olfactory receptor neurons (ORNs) are at the front end of the olfactory system. Each ORN expresses only one type of odorant receptor (OR). ORNs of the same type converge into the same glomerulus of the antennal lobe in insects (or the olfactory bulb in humans)^1^,^2^,^3^,^4^,^5^,^6^,^7^,^8^,^9^.

The olfactory system uses a combinatorial code. Unlike many other receptors that are activated by only one specific ligand, such as a neurotransmitter or a hormone, an OR can be triggered by many odorant molecules. Furthermore, an odorant molecule can interact with different types of OR^10^. The combinatorial code enables humans to discriminate many odors^11^ by using a repertoire of only approximately 350 ORs. However, it is not yet clear which properties of a molecule contribute to its smell. This question is a topic of ongoing research, and many theories have been proposed^12^,^13^,^14^,^15^,^16^,^17^,^18^,^19^,^20^,^21^,^22^,^23^,^24^,^25^,^26^.

Odorant receptors are transmembrane proteins, and in vertebrates, they are metabotropic receptors that belong to the G-protein coupled receptor (GPCR) family^27^,^28^. In insects, the signaling methods of ORs are a topic of debate. Insect ORs are thought to be ionotropic receptors but may also use metabotropic signaling^29^,^30^,^31^,^32^,^33^. The topology of ORs in insects is different from that in vertebrates^34^,^35^, and most insect ORs function in the presence of another common receptor known as Orco^36^.

Many similarities exist between the olfactory system of insects and that of vertebrates^37^,^38^. Regardless of the signal transduction pathway utilized, all ORs have the same function: they have a binding pocket (also known as a binding cavity or a binding site), where odorants (also known as ligands) bind. Binding to an odorant activates an OR, and the activated OR changes the potential of the cell either directly (ionotropic) or indirectly (metabotropic); therefore, knowledge regarding the olfactory system of *Drosophila* could potentially help us to decode human olfaction.

The amplitude of the change in the membrane potential of an ORN depends on the number of activated ORs and the duration of their activation, which are both determined by various physicochemical properties of the odorant and the OR^12^,^14^,^18^,^39^,^40^. One important factor is the size of the ligand relative to the OR binding pocket. Another factor is the flexibility of the binding pocket. Proteins are not rigid bodies and can change shape depending on the amino acids involved^41^,^42^,^43^. The size and flexibility of binding pockets have been used in computational drug design to predict the binding pocket of a given ligand^44^.

Herein, we focused on the volume and flexibility of the binding pocket. The molecular volume of a ligand should match the dimensions of the OR binding pocket. Subsequently, the ligand can fit into the binding pocket of the OR and trigger signal transduction. Mismatches in volume decrease the neural response; however, flexibility of the binding pocket can compensate for volume mismatches (Fig. 1).

We can determine the volume and flexibility of a binding pocket if we know its three-dimensional structure. However, the structures or ORs are unknown because it is difficult to determine the structure of integral membrane proteins^45^,^46^. To investigate OR protein structure, various research methods have been used, including molecular dynamics (MD) simulations, mutagenesis studies, heterologous expression studies, and homology modeling^47^,^48^,^49^,^50^,^51^,^52^,^53^,^54^,^55^.

In the current study, we develop a mathematical framework that utilizes available experimental data, and we apply this developed mathematical framework to investigate the relationship between the molecular volume of odorants and the ORN response. Our results suggest that although molecular volume is a considerable factor, it is not the only factor that determines the neural response of ORNs. We predict the *in vivo* volumes and flexibilities of OR binding pockets (supplemental file volume-profiles.csv) by applying our mathematical method to neural data from the Database of Odorant Receptors (DoOR)^56^, which is a well-structured database that includes the neural responses of most *Drosophila* ORs to many odorants^56^. This database aggregates data from many sources^17^,^19^,^57^,^58^,^59^,^60^,^61^,^62^,^63^,^64^,^65^,^66^,^67^,^68^,^69^.

We suggest that a functional relationship exists between molecular volume and the neural response. We also provide a methodology to estimate the *molecular receptive range* or *tuning function* of ORs. Finally, we predict the structural properties (i.e., volumes and flexibilities) of OR binding pockets. Our results may aid in the selection of odorants for future experimental studies (supplemental file proposed-odorants.csv) and may contribute to the study of olfactory coding by unmasking the effects of other possible factors.

## Material and Methods

We used the neural data of the DoOR 1.0^56^ database for our calculations, and we reserved the additional data in the DoOR 2.0^18^,^70^,^71^,^72^,^73^,^74^,^75^ database to use as a test set. We calculated the molecular volume (supplemental file odorants.csv) using the computational chemistry software VEGA ZZ^76^. We used GNU R statistical computing software to analyze the data^77^.

The DoOR database includes an *N* × *M* matrix. Its elements, *r*_*nm*_, are the response of ORN *n* to odorant *m*. This matrix is normalized to have values between 0 and 1, so 0 ≤ *r*_*nm*_ ≤ 1, where 1 is the strongest response. This matrix has many *Not Available* (NA) values, and different ORNs are excited by different sets of odorants. We accounted for this feature by removing NA values from the summations and calculating 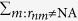; however, for brevity, we used the usual notation 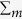.

The response *r*_*nm*_ may depend on the molecular volume of the odorant, *v*_*m*_, and other physicochemical properties of the molecule *m*; therefore, we separated the response *r*_*nm*_ into two terms:


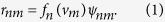


**Figure 1:**
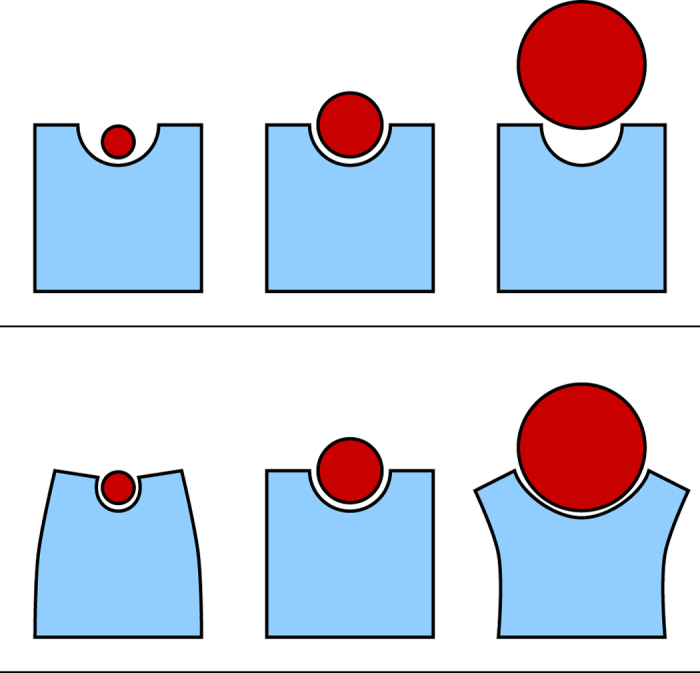
This figure shows different scenarios that may occur when an odorant molecule (ligand) binds to an odorant receptor according to the coarse-grained model. The red disks represent the odorant molecule, and the blue shapes represent the odorant receptor (OR) and binding pocket. The top schematic shows a mismatch because of the small molecular volume on the left, a perfect match in the center and a mismatch because of a large molecular volume on the right. The bottom schematic shows how the flexibility of an OR may compensate for molecular volume mismatches.

The first term, *f*_*n*_(*v*_*m*_), depends only on the molecular volume of the odorant. The second term, the volume-independent term ψ_*nm*_, includes every other influential property of the odorant molecule, with the exception of molecular volume or any other property that correlates with molecular volume (e.g., molecular weight). Of the molecular parameters that correlate with molecular volume, we used molecular volume because it fits the acceptable picture of protein-ligand interaction (Fig. 1). Using molecular weight would have implied receptors use some type of mass spectroscopy analysis. We tested a few other important parameters, including polarity, functional group, and polar surface area; however, none of the parameters were as dominant as molecular volume. Therefore, we primarily focused on molecular volume (*f*_*n*_(*v*)) and may consider other parameters (*ψ*_*n*_*m*) in future studies.

Each of the two terms was characteristic of the OR and varied for each OR. In fact, the first term, *f*_*n*_(*v*), can be considered to be the tuning curve of an ORN *n* with respect to the molecular volume. We approximate this term with a Gaussian function,


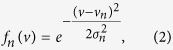


where *v*_*n*_ is the preferred molecular volume of the OR *n*, and *σ*_*n*_ represents the flexibility of the OR binding pocket. We used a Gaussian function for the tuning curve for the following reasons: (a) it is among the simplest forms that can describe a preferred volume and flexibility, and (b) the mathematics was easy to follow and the final solution was simple.

In this work, we wanted to estimate *v*_*n*_ and *σ*_*n*_. Thus, we first calculated the response-weighted average of the molecular volumes, 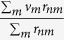, and then we used (1):


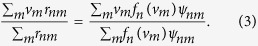


We approximated ∑ with ∫, which is common in statistical physics:


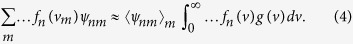


**Figure 2:**
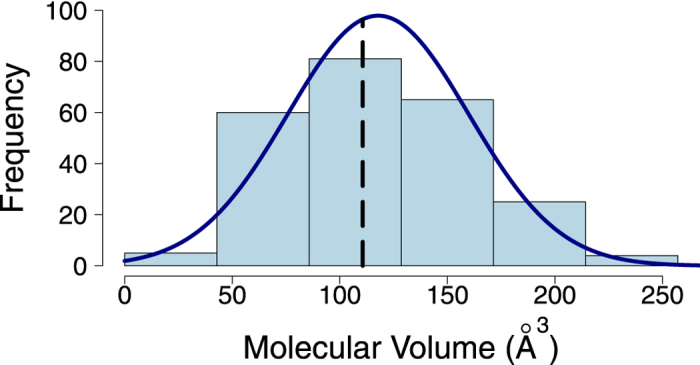
The graph shows the density function of molecular volumes, *g*(*v*), for all molecules in the DoOR database. The solid line is a Gaussian fit (Eq. 5), and the dashed line shows the median, which is slightly different from the mean.

In this equation, 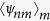 denotes the average of *ψ*_*nm*_ over all 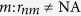. We moved 〈*ψ*_*nm*_〉_*m*_ out of the integral because it is independent of *v*. Here, *g*(*v*) is the density of states, and *g*(*v*)*dv* indicates how many molecules have a molecular volume in the range of *v* and *v* + *dv*. This function was approximated by a Gaussian function (Fig. 2),


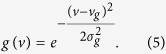


Ideally, *g*(*v*) must not depend on the OR *n* because it is a property of the ensemble of odorant molecules and not a property of the OR. We also had many missing values (*r*_*nm*_ = *NA*) that did not overlap, and we had to calculate *g*(*v*) for each ORN separately; therefore, 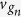 and 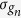 are the average and standard deviation, respectively, of the molecular volume while *r*_*nm*_ ≠ NA. We rewrote equation (3) using equation (4):


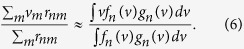


To obtain a simpler form, we replaced the product of *f*_*n*_(*v*) and *g*_*n*_(*v*) in the above equation with *h*_*n*_(*v*) = *f*_*n*_(*v*)*g*_*n*_(*v*).


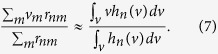


The function *h*_*n*_(*v*) is a Gaussian function because it is the product of two Gaussian functions,


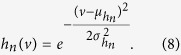


Thus, the right side of equation 7 was nothing but 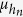, and in a similar manner, we calculated 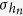 from the neural data.


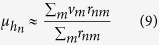



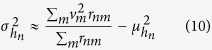


We know the mean, 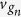, and standard deviation, 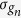, of *g*_*n*_(*v*) from the molecular volumes of the ensemble of odorants. We calculated the mean 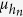 and standard deviation 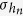 of *h*_*n*_(*v*) from the neural data. Using these values, we calculated the mean *v*_*n*_ and the standard deviation *σ*_*n*_ of *f*_*n*_(*v*). First, we calculated *σ*_*n*_ using


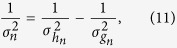


and then we calculated *v*_*n*_:


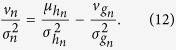


**Figure 3:**
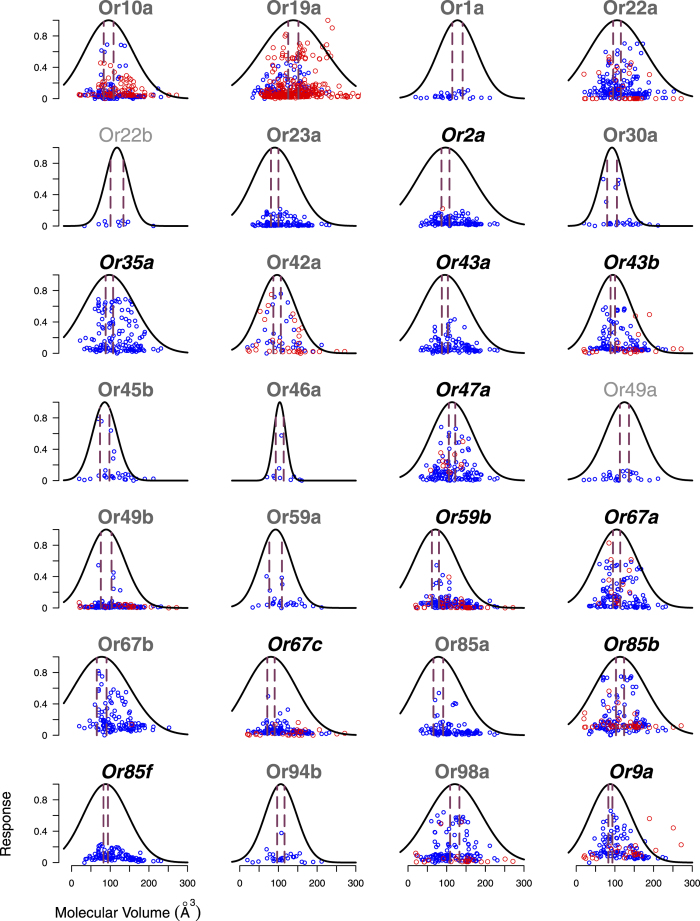
The response of ORs versus the molecular volume of odorants (circles). The fitted functions *f*_*n*_(*v*) from Eq. 1 (solid lines) and the error bars of the mean of *f*_*n*_(*v*) (red vertical lines) for 28 ORs showed that their responses were significantly dependent (p-value < 0.05) on molecular volume. Except 2 (ORs name in light gray), 26 were significant according to the FDR correction (ORs named in gray), and 11 were significant according to the Bonferroni correction (ORs with names in black). The function *f*_*n*_(*v*) was calculated based on data from the DoOR 1.0 database (blue circles). The red circles are additional data from the DoOR 2.0 database.

The calculated *v*_*n*_ and *σ*_*n*_ are provided in the supplemental file volume-profiles.csv. The resulting *f*_*n*_(*v*) are plotted over the actual data for the 28 ORs (Fig. 3) in which the p-values were <0.05.

We calculated p-values using permutation tests and shuffled the data 10^5^ times. We shuffled the association between odorants and the responses of a given OR and then checked the null and alternative hypotheses. The alternative hypothesis was that “ *the response of the ORN depends on the molecular volume of the odorant*”, which requires a finite value for *σ*_*n*_. The null hypothesis was that “ *the response of the ORN is independent of the molecular volume of the odorant*”, which requires *σ*_*n*_ → ∞. Therefore, the p-value is the probability of having 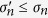, where *σ*_*n*_ is calculated from the original data, but 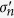 is calculated using the permuted version.

**Figure 4:**
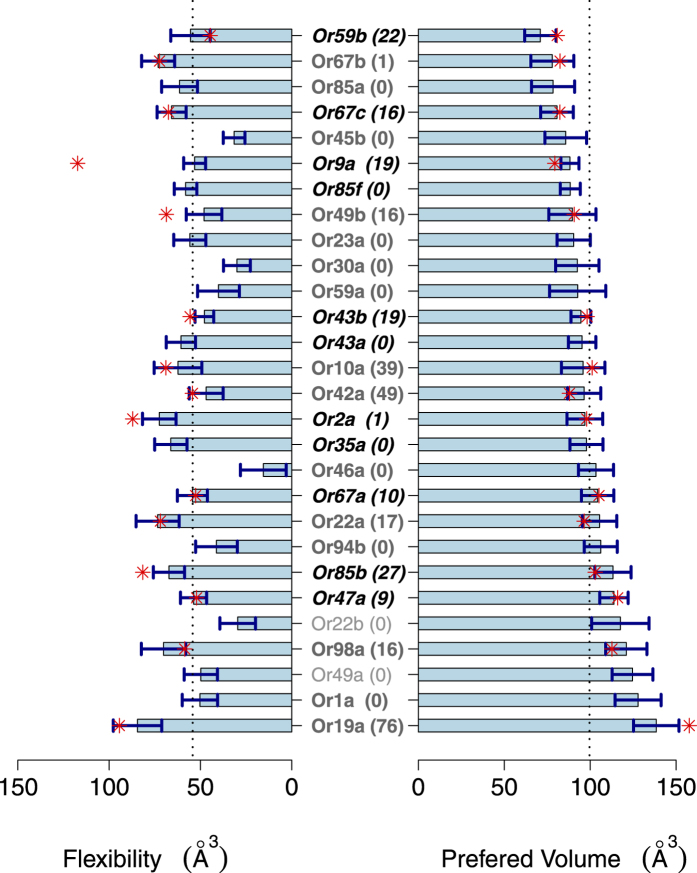
The preferred volumes *v*_*n*_ (right) and flexibilities *σ*_*n*_ (left) of 28 ORs. The error bars were calculated using the Jack-Knife method. Some ORs, including Or59b, Or67a and Or85a, preferred smaller molecules, but some ORs, including Or19a, Or1a and Or49a, preferred larger molecules. Some ORs, such as Or46a, Or22b and Or30a, were volume selective, but other ORs, including Or19a, Or67b and Or22a, responded to a broader range of molecular volumes. Asterisks indicate the updated results using the DoOR 2.0 database, and the numbers in parentheses show the percentage of DoOR 2.0 results relative to the total amount of data for each receptor.

We tested the hypotheses on ~60 ORs simultaneously (only 44 were present in the DoOR 1.0 database). Using a simple threshold of 0.05 for the p-value of each OR would have resulted in many false positives. To address the issue of a multiple-comparison problem, we used the Bonferroni correction (by multiplying the p-values by 60). The problem with the Bonferroni correction is that it may increase the number of false negatives. This problem can be addressed by using another method called the false discovery rate (FDR) that keeps the rate of false positives below a threshold^78^,^79^. We used the Bonferroni and FDR methods as well as no correction. We used the function *p.adjust* of GNU R to calculate the corrected p-values. The results were labeled accordingly in Figs 3 and 4.

**Figure 5:**
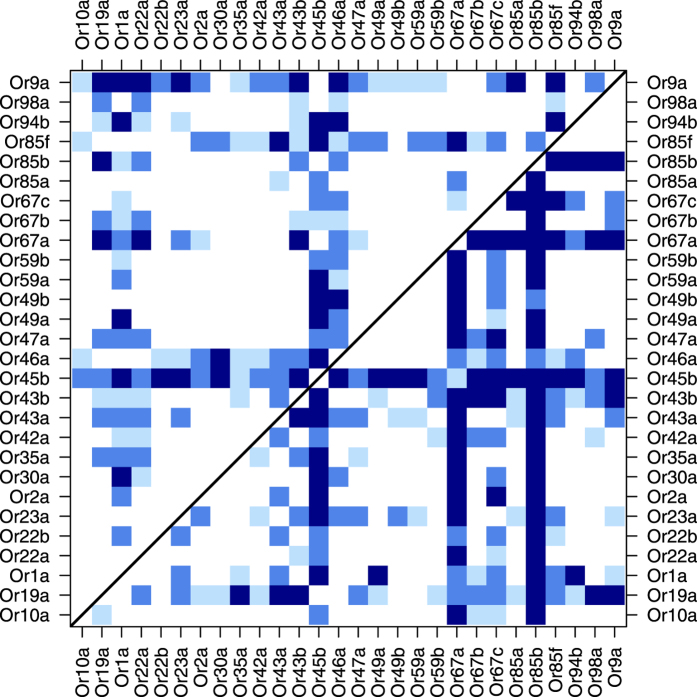
Pairs of ORs that differed significantly in their binding-pocket volumes (upper triangle) and flexibilities (lower triangle). All blue shades indicate a p-value less than 0.05. The two darker shades indicate FDR-corrected p-values less than 0.05, and the darkest shade has a Bonferroni-corrected p-value less than 0.05.

We also wanted to show the diversity of volumes and flexibilities of binding pockets among ORs. To estimate the p-values, we used any pair of ORs that were sensitive to molecular volume (28 ORs), calculated their difference, used a permutation test (6 × 10^4^ shuffles) and measured the probability of obtaining different results (Fig. 5).

## Results and Discussions

The relationship between molecular volume and the ORN response was evident (Figs 3, 4, 5). The function *f*_*n*_(*v*) was considered to be the tuning curve of OR *n* in response to molecular volume (Fig. 3). Each OR had a preferred molecular volume *v*_*n*_ and showed some flexibility *σ*_*n*_. The calculated *f*_*n*_(*v*) values are shown in Fig. 3. This figure includes 28 ORs that showed a significant dependence on odorant molecular volume in their response (p-value < 0.05).

The flexibility of a receptor may affect the broadness of its tuning curve (flexible receptors may bind to more odorants), but we did not see any significant relationship when using three definitions of broadness: depth of selectivity, breadth of selectivity and kurtosis^70^,^80^,^81^.

The results of 28 ORs indicated that 11 ORs were significant according to the Bonferroni correction (ORs with black names), 26 of them were significant according to FDR correction (ORs with gray names), and the remaining receptors (2 ORs with light gray names) only satisfied the criteria of a p-value < 0.05 without any corrections. After applying the FDR correction, more than half of the available ORs in the DoOR 1.0 database (26/44) showed significant sensitivities toward molecular volume. The remaining receptors may be sensitive to molecular volume as well; however, the current evidence is not sufficient, and more experiments are necessary.

**Figure 6:**
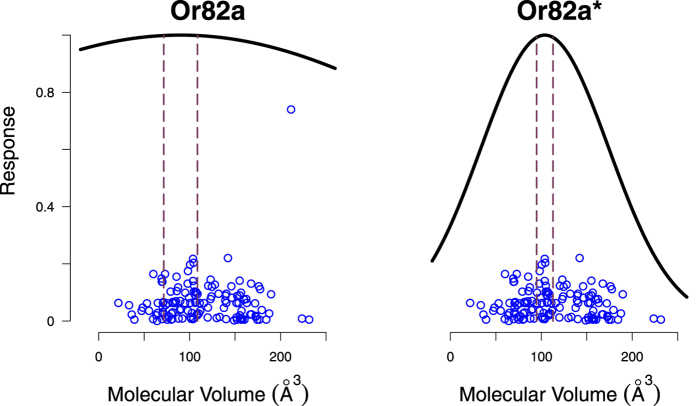
The response of Or82a to odorants. Geranyl acetate (the outlier) did not confirm our theory and had a p-value of 0.55 (left); however, when geranyl acetate was removed from the data, Or82a confirmed our model with a Bonferroni-corrected p-value of 0.03 (right).

One interesting case in this regard was Or82a, which did not fit our hypothesis. Or82a binds to geranyl acetate much better than to any other molecule. When we removed geranyl acetate from the data, suddenly Or82a fit perfectly to our model with a Bonferroni-corrected p-value of 0.03 (Fig. 6). The underlying interaction between geranyl acetate and Or82a is therefore a special case that requires more investigation.

The parameters of *f*_*n*_(*v*), *v*_*n*_ and *σ*_*n*_ are shown in Fig. 4. Figure 4 demonstrates that the molecular volume preferences of ORs were different (right), and the flexibilities of the ORs were also different (left). To support these claims, we estimated the p-values of having different volume preferences and flexibilities for each pair of 28 ORs (Fig. 5). The comparison of the volume preferences of all 378 possible pairs indicated that 133 had a p-value less than 0.05. This number was reduced to 89 after using the FDR correction and further reduced to 32 after using the Bonferroni correction. The corresponding number of pairs with a p-value less than 0.05 was 168, 134 and 77, respectively, for the flexibility comparisons. The union of these two sets confirmed that 226 (p-value < 0.05), 171 (FDR corrected), and 91 (Bonferroni corrected) pairs of ORs showed distinct differences in their binding-pocket characteristics.

The diversity of ORs is important in perceiving the quality of smells. In a hypothetical experiment, assume that all odorant molecule characteristics are the same with the exception of molecular volume. If all ORs have the same preferred volume and flexibility, any change in the molecular volume will change only the intensity of smell and not its quality. Here, we showed that ORs have different preferred volumes and flexibilities. Therefore, any change in the molecular volume of an odorant results in a different combinatorial encoding, which affects the quality and intensity of the perceived smell. This conclusion is in agreement with the work of M. Zarzo that suggested that larger molecules smell better^82^ and might account for differences between the scents of methanol, ethanol, propanol and butanol. Methanol smells pungent, ethanol smells pleasant and wine-like, and propanol and butanol smell like ethanol; however, butanol has a slight banana-like aroma. We argue that molecular volume affects combinatorial encoding and that combinatorial encoding determines odorant quality.

Herein, we showed that the responses of ORNs are related to odorant molecular volume. However, it is not clear what other features of molecules are measured by ORs. Many studies have attempted to connect the physicochemical properties of molecules to the evoked neural response and/or the perceived smells; however, the nonlinear volume dependence (Eq. 1 and Eq. 2) may mask important correlations between molecules and neural responses. When *f*_*n*_(*v*) is close to zero, the value of *ψ*_*nm*_ does not matter.

We predicted that odorants with a molecular volume in the tail regions of *f*_*n*_(*v*) remain undetected, regardless of any of their other physicochemical properties. This prediction can be confirmed in future experiments.

**Figure 7:**
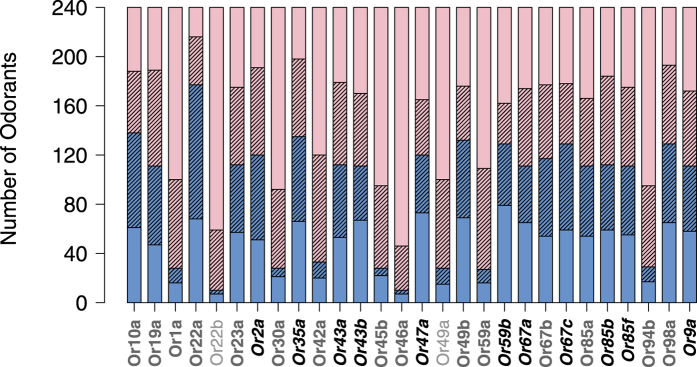
Venn diagram of the DoOR 1.0 database and our suggested important odorants for each OR. The database includes 240 molecules. Some of the 240 molecules have been used to study an OR (blue areas); however, data for the rest of the molecules are not available for some of the ORs (pink). The hatched areas represent odorants with molecular volumes that are close to the preferred volume of each OR 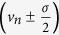. We already know the neural responses of the hatched blue areas, but the hatched pink odorant areas can be the target of future experiments. We predict that the remaining odorants will only yield no response.

When studying the *ψ*_*nm*_ of an OR, it is better to have many data points, and it is better for the data points to be close to the preferred volume of the OR; however, the current data do not meet these conditions. For many ORs, most data points are in the tail regions of *f*_*n*_(*v*), with values close to zero. We have included the best selection of odorants for each of the 28 studied ORs (see Venn diagram in Fig. 7 and supplemental file proposed-odorants.csv); this information can be used to save time and expenses during future experiments.

We have also predicted some *in vivo* structural aspects of OR binding pockets: the preferred volume of each OR results from the volume of the binding pocket, and the flexibility of an OR results from the rigidity or flexibility of the binding pocket. These data provide additional constraints on the 3D structure of ORs, which may aid in the prediction and calculation of the 3D structure of these proteins.

The methods of the current study can also be combined with mutagenesis. When an OR gene is mutated, the response to a selection of molecules can be subsequently measured, and finally, the preferred volume and flexibility can be calculated. In this way, we could potentially understand which amino acids affect the function of the OR and contribute to both the volume and flexibility of the binding pocket.

In this manuscript, we have excluded many factors because the nature of the problem is inherently complex; it would not be feasible to study this problem with all possible factors. Many factors affect the concentration of odorant molecules at ORs, including the molecular mass, the method of mixing odorants and air, the vapor pressure, the solubility of odorants in water, the sensillum lymph and odorant-binding proteins (e.g., LUSH)^83^,^84^. It is difficult to control for all of the aforementioned factors in the current experimental paradigm, and the model would be very complex with many sets of parameters. For example, if we introduce an odorant into air, there will be a mixture of air, vapor and mist. Then, the mixture reaches the sensilla, mixes with sensillum lymph fluid, may bind to odorant-binding proteins and finally reaches ORs. Two important parameters in this process are vapor pressure and water solubility. Vapor pressure limits the vapor concentration of a liquid. Water solubility limits the amount of odorant that can dissolve in water. Both factors are nonlinear at high concentrations; therefore, we can neglect the effect of vapor pressure and water solubility. However, if we are close to the critical concentrations, vapor pressure and water solubility are very important.

We expect these factors to have minimal effects on smaller molecules because they evaporate easily, readily dissolve in water and might not need the help of odorant-binding proteins. Therefore, we have greater confidence about the lack of response to small molecules than we do about the lack of response to larger molecules. Using an experimental paradigm similar to a luciferase assay^85^ may provide valuable complementary information to our simple model. When using a luciferase assay, the concentrations are accurate, but the experiment is *in vitro*.

## Conclusion

We showed that molecular volume is an important factor, but it is not the only factor that determines the response of ORNs.

We hypothesized that the ORN response results from OR binding-pocket volume and flexibility. We predicted the actual *in vivo* volumes and flexibilities of OR binding pockets. The results are provided in supplemental file volume-profiles.csv, and they can be verified when the 3D structures are resolved and/or when more experimental results are available.

Now that we understand the extent to which molecular volume contributes to the ORN response, it is possible to study the effects of other parameters.

We approximated a molecule as a rigid isotropic sphere of a given volume, but our model does not consider the shape^13^,^14^,^40^, vibrational mode^12^,^16^,^24^, chirality^86^ or many other potentially interesting properties of a molecule. Our methods and results actually provide a starting point that may lead to the study of other factors.

An improvement to this model would be to include the anisotropy of the molecules by modeling them as ellipsoids. This modeling will capture more aspects of the molecular shape and may aid in the inclusion of constitutional isomers.

Approximating *f*_*n*_(*v*) and *g*(*v*) with a Gaussian function makes the mathematical formulation simple and readable. However, a semi-infinite function may be a better choice for molecular volumes, which cannot have negative values.

Although this work utilized data from *Drosophila*, we expect that the general principles and methodologies of this work will also apply to vertebrates. We are working to apply the same method to human odorant receptor data^85^.

## Additional Information

**How to cite this article**: Saberi, M. and Seyed-allaei, H. Odorant receptors of *Drosophila* are sensitive to the molecular volume of odorants. *Sci. Rep.* **6**, 25103; doi: 10.1038/srep25103 (2016).

## Supplementary Material

Supplementary Information

Supplementary Information

Supplementary Information
